# Implication of Obesity and Gut Microbiome Dysbiosis in the Etiology of Colorectal Cancer

**DOI:** 10.3390/cancers15061913

**Published:** 2023-03-22

**Authors:** Samradhi Singh, Poonam Sharma, Devojit Kumar Sarma, Manoj Kumawat, Rajnarayan Tiwari, Vinod Verma, Ravinder Nagpal, Manoj Kumar

**Affiliations:** 1 Indian Council of Medical Research-National Institute for Research in Environmental Health, Bhopal 462030, India; 2 Stem Cell Research Centre, Sanjay Gandhi Post-Graduate Institute of Medical Sciences, Lucknow 226014, India; 3 Department of Nutrition and Integrative Physiology, Florida State University, Tallahassee, FL 32302, USA

**Keywords:** colorectal cancer, obesity, gut microbiota, inflammation, probiotics, FMT, prebiotics, bacteriophage therapy, gut dysbiosis, biofilms

## Abstract

**Simple Summary:**

Obesity is associated with an increased risk of colorectal cancer (CRC). Recent studies suggest that gut dysbiosis, i.e., abnormal perturbations in the gut microbiome (the highly diverse and complex community of microorganisms inhabiting our gastrointestinal tract) may play a crucial role in this obesity–CRC link. This microbiome imbalance can lead to alterations in the metabolism of the microbiome that can promote cancer development. Therefore, understanding the role of obesity and associated gut dysbiosis can help in identifying novel strategies for the prevention and treatment of CRC.

**Abstract:**

The complexity and variety of gut microbiomes within and among individuals have been extensively studied in recent years in connection to human health and diseases. Our growing understanding of the bidirectional communication between metabolic diseases and the gut microbiome has also highlighted the significance of gut microbiome dysbiosis in the genesis and development of obesity-related cancers. Therefore, it is crucial to comprehend the possible role of the gut microbiota in the crosstalk between obesity and colorectal cancer (CRC). Through the induction of gut microbial dysbiosis, gut epithelial barrier impairment, metabolomic dysregulation, chronic inflammation, or dysregulation in energy harvesting, obesity may promote the development of colorectal tumors. It is well known that strategies for cancer prevention and treatment are most effective when combined with a healthy diet, physical activity, and active lifestyle choices. Recent studies also suggest that an improved understanding of the complex linkages between the gut microbiome and various cancers as well as metabolic diseases can potentially improve cancer treatments and overall outcomes. In this context, we herein review and summarize the clinical and experimental evidence supporting the functional role of the gut microbiome in the pathogenesis and progression of CRC concerning obesity and its metabolic correlates, which may pave the way for the development of novel prognostic tools for CRC prevention. Therapeutic approaches for restoring the microbiome homeostasis in conjunction with cancer treatments are also discussed herein.

## 1. Introduction

Colorectal cancer (CRC) is the third most common cancer, with an estimated one million deaths related to it each year, making it the second most frequent cause of cancer-related mortality worldwide [1]. According to the IARC (International Agency for Research on Cancer), this number is projected to increase by 56% between 2020 and 2040 [1]. An estimated 69% increase in disease-related fatalities is predicted, resulting in around 1.6 million deaths worldwide in 2040 [1]. The incidence of cancer is likely to keep increasing due to the rise in risk factors, particularly obesity and metabolic syndromes. Excess energy consumption and a lack of physical activity have been related to a sharp rise in the prevalence of obesity. Obesity is indicated by a BMI of ≥30 kg/m^2^, and almost all developed and developing countries are now experiencing a sharp increase in the incidence of overweight (BMI ≥ 25 kg /m^2^) and obesity, which affects up to 70% of adults in industrialized nations and is more common in women and urban regions [2]. Particularly in recent decades, obesity has escalated into a global epidemic and public health crisis, and its prevalence is still increasing at a greater frequency. According to the most recent NCD (Non-Communicable Diseases) Risk Factor Collaboration data, about 2 billion adults (39% of the world’s adult population) were predicted to be overweight in 2016, with 671 million (12% of the world’s adult population) obese, a threefold increase in obesity since 1975 [3]. If this trend continues, one billion adults could be obese by 2025 [4]. Obesity is linked to an increased risk of premature death and is a major contributor to the global burden of noncommunicable diseases, including cancer [4,5]. An analysis of the population-attributable percentage reveals that obesity is linked to 11.9% of cancer cases in men and 13.1% in women [2]. Numerous epidemiological studies demonstrate that adult obesity increases the risk of colon cancer by 1.2 to 2 times, with obesity thought to be responsible for between 14% and 35% of all cases of this cancer [6,7,8]. Ectopic fat tissue, which forms due to excessive or aberrant fat tissue deposition, that exceeds the epigenetically and genetically determined adipose tissue stores can increase the risk of developing CRC due to its effect on metabolic pathways and inflammatory processes [9]. Adipose tissue is distributed throughout the body in different compartments, each with a distinct role depending on the bodily location. Visceral adipose tissue, found mainly in the abdominal cavity around organs such as the liver, pancreas, and intestines, has been linked to an increased risk of developing CRC in several studies [10,11,12]. Unlike overall obesity, as measured by BMI, visceral obesity (VO) may be a more significant risk factor for CRC due to its metabolic implications. Visceral fat produces hormones such as adipokines, cytokines, and reactive species that promote inflammation and insulin resistance, both of which are associated with CRC development [13,14]. Additionally, obesity presents worse health effects when paired with sarcopenia, which is defined as the loss of skeletal muscle mass and strength. Sarcopenic obesity (SO) is caused by a number of endocrine-hormonal, metabolic, and lifestyle factors that, in turn, influence pathophysiological factors that may aid in the development of cancer [15,16]. Cancer also involves inflammatory components, such as the imbalanced production of inflammatory cytokines and reactive oxygen species, which are linked to both obesity and sarcopenia. Oncogenesis and SO are mediated by factors including extracellular matrix remodelling, type-2 diabetes, gut microbiota dysbiosis, immune system dysfunction, imbalance of adipokines and myokines, and insulin resistance [15,16,17]. Together, these phenomena correlate with the incidence of CRC development and progression. In addition to increasing the risk of CRC, both VO and SO have a negative impact on CRC prognosis and may have negative clinical implications in CRC patients such as higher recurrence rates, higher risk of dose-limiting toxicity or chemotherapy toxicity, surgical complications, delayed recovery after surgery, physical disability, shorter survival, and a greater risk of developing metastasis [18,19,20,21,22,23]. These implications might serve as a valuable point of reference for the prognostic significance of SO and VO. According to a dose-response meta-analysis, a body weight rise of 10 kg was associated with an approximately 8% higher risk of CRC [24]. Obese people in their youth have a higher risk of acquiring CRC than adults. As expected, a reduction in body weight through bariatric surgery results in a 27% lower incidence of CRC [24]. To understand the cellular and molecular processes at play in the obesity–cancer relationship is a vital step towards improving cancer prevention and treatment approaches. The relationship between obesity and cancer is a complicated one, with various factors influencing the development of the disease. Obesity-induced abnormal lipid metabolism, altered levels of adipokines and hormones, dysbiosis of the gut microbiota, chronic inflammation, and altered bile acid balance may all contribute to the intricate metabolic regulation of CRC carcinogenesis. The gut microbiome is constantly adapting to its human hosts, and changes in the last few decades are thought to be linked to an increase in obesity. Since the gut microbiota is known to play a significant part in regulating host metabolism, any alteration in the composition of the gut microbiota or the levels of its metabolites can affect a variety of physiological processes, including inflammation, energy metabolism, cell proliferation, and apoptosis, which are important factors in CRC development and progression. In these contexts, this review contemplates and discusses the role of obesity and gut microbiome dysbiosis in CRC etiology and pathophysiology, highlighting the fundamental biological mechanisms that underlie this association as well as recent advances that offer novel insights into pathogenetic mechanisms. In addition, potential therapeutic intervention approaches that aim to regulate the gut microbiome and microbial metabolic pathways that promote the rise of obesity and associated CRC are also discussed.

## 2. Obesity and Gut Microbiome Dysbiosis: An Intricate but Established Association

Our awareness of the gut microbiota as a factor that directly impacts our health or illness status has increased during the past decade. It has been specifically connected to the development of obesity. In fact, the makeup of gut bacteria seems to influence obesity [25]. The gut microbiome is an intricate and complex system that consists of an estimated 100 trillion microorganisms and contains nearly 200 times more genes than the human genome. This enormous variety of bacteria can be considered an independent “organ” [26]. *Firmicutes* and *Bacteroides* make up 90% of the bacteria in the gut of healthy adults, with *Actinobacteria* (mostly *Bifidobacterium*), *Proteobacteria, Fusobacteria*, and *Verrucomicrobia* making up the remaining 10% or less [27]. The gut microbiota’s composition varies significantly among individuals and even alters over time. The co-evolution of humans and their microbiota has resulted in biomolecular networks between them because of their mutual dependence on one another for survival. In this condition, bacterial populations are dynamic and susceptible to changes in the host’s environment and body [26]. To sustain its high population levels, the intestinal microbiota relies on food that is not digested by the human body, dead cells that act as nutrients, and the mucus secreted by the gut [28]. These microbes perform key functions such as producing short chain fatty acids (SCFAs), biodegrading polysaccharides, enriching specific lipopolysaccharide (LPS), and synthesizing essential amino acids and vitamins. The gut microbiota also generates a wide range of physiologically active molecules with beneficial properties, such as anti-inflammatory, antioxidant, and analgesic effects, as well as potential harm including carcinogens, neurotoxins, and immunotoxins. These substances can enter the bloodstream and alter gene expression, impacting metabolic and immunological processes [29,30]. Thus, a balanced gut flora is essential for maintaining metabolic balance and energy levels. Additionally, dynamic equilibrium allows the microbiota to resist disruption and return to its healthy state, such as after antibiotic therapy. Research has also uncovered a connection between gut microbiota composition and weight, as it can influence nutrient absorption and energy expenditure. Unbalanced gut microbial populations can lead to an increased intake of calories, increased storage of body fat, and metabolic issues resulting in obesity [31,32] (Table 1). It has also been linked to changes in hormones that regulate metabolism and appetite, leading to a greater risk of obesity. Through its impact on adiposity and glucose metabolism, the gut microbiota plays a significant role in the start, progression, and comorbidities of obesity [33]. Additionally, dysregulated autophagy activity, AMPK and PGC-1α signaling, and gut dysbiosis may be greatly implicated in the pathological link between SOB and related complications [17]. In addition, the gut microbiota can also play a role in regulating the immune system. This can lead to increased inflammation, which is associated with obesity. Recent research has shown that changes in the gut microbiota (at phylum or species level) as well as intestinal inflammation are related to weight gain and the metabolic effects of obesity [31]. Compared to healthy individuals, obese patients’ gut microbiota are less diverse [34]. The ratio of *Firmicutes* to *Bacteriodetes,* the *Enterobacteriaceae* species, and the *Bacteroidales* genera including *Bacteroides* spp., *Lactobacillus* spp., *Enterococcus* spp., and *Bifidobacterium* spp. are all upregulated in obesity, while *Clostridia*, including *Clostridium leptum*, and *Enterobacter* spp., are downregulated [35,36,37,38,39,40,41,42]. Sterile mice models lacking gut flora are protected from obesity, even after feeding a high-fat diet (HFD) [43]. Additionally, lean germ-free mice were injected with the intestinal microbiota of obese mice or women. These mice subsequently gained body fat and had metabolic problems linked to obesity [44,45]. Fecal microbiota transplantation (FMT) from lean to obese subjects improves insulin sensitivity in the recipients while also causing acute changes in the gut microbiota in humans [46]. Gut microbiota changes that may promote obesity and its associated comorbidities include appetite regulation via the gut–brain axis, various hormones, and neuromodulators, increasing host energy absorption by altering gene expression and levels of SCFAs, regulating fat storage through lipoprotein lipase and transcription factors, chronic inflammation brought on by LPS and inflammatory gene expression, disrupting the circadian rhythm by affecting the circadian transcription factors, epigenetic modifications, increasing fat accumulation by decreasing liver fatty acid oxidation by suppressing the adenosine monophosphate kinase (AMPk), and increasing the synthesis of toxic metabolites such as secondary bile acids (SBAs) [32,34]. These alterations are closely related to visceral fat accumulation; therefore, targeting specific microbial species and pathways closely associated with visceral fat accumulation might lead to new therapies for obesity and associated disorders [47]. Due to the richness and complexity of the gut microbiota, further research is still needed to determine the precise mechanism by which it causes obesity.

## 3. Potential Mechanisms Linking Obesity, Microbiome, and CRC

Under normal circumstances, the gut microbiota is essential for maintaining the normal physiological function of the digestive tract, promoting food digestion and absorption, and regulating immunity. Dysbiosis is characterized by changed microbiota composition, bacterial bioactivity, and distribution in various regions of the human body. Dysbiosis occurs when the gut microbiota is influenced by multiple factors, including the diet, environment, and host genes. These changes can lead to diseases such as cancer, inflammatory bowel disease (IBD), cardiovascular disease, type 2 diabetes, and mental disorders. Despite accumulating evidence that there is a favorable correlation between obesity and CRC, the underlying molecular pathways remain poorly understood. The intricate metabolic regulation of CRC carcinogenesis may be significantly influenced by obesity-induced dysbiosis of the gut microbiota. Pharmacological interventions, such as taking antibiotics, host illnesses such as infectious diarrhea, and dietary factors, such as high-fat and low-fiber diets, can all lead to microbial dysbiosis, which alters the gut microbiota and its functioning and may trigger abnormal host immune responses. Human obesity, which is characterized by the decline in gut microbial diversity, is connected to the dysregulation of metabolism which leads to the development of additional disorders [71,72,73].

Intriguingly, the colon has one million times more bacteria than the small intestine, and the former has about 12 times more malignancies than the latter, suggesting that the gut microbiota may play a role in colorectal carcinogenesis [74]. Although the mechanisms underlying dysbiosis and changes in microbial richness are poorly understood, it is unclear whether dysbiosis is a cause or a result of CRC. A dysbiotic microbial community with pro-carcinogenic features can remodel the microbiome as a whole to drive pro-inflammatory reactions and epithelial cell transformation, which leads to cancer. In fact, the CRC microenvironment is characterized by host-derived immunological and inflammatory responses that may have an impact on microbial regulation, change the composition of the microbiota, and encourage the growth of particular bacteria that may have cancer-causing effects [75]. With the emergence of “keystone pathogens” that have significant impacts on bacterial composition and consequently promote dysbiosis, dysbiosis in CRC may thus result in the selection of microbiota composition via a tumor-linked microenvironment [53]. The altered gut microbiota of colon adenoma patients suggests dysbiosis may contribute to the early stages of CRC development [76,77]. According to another study, mice fed an HFD were more likely to develop CRC due to obesity-induced intestinal dysbiosis. They discovered that CRC formation and progression might be accelerated by simply transplanting the feces of obese Kras (G12Dint) mice to mice of normal weight [78]. The question of whether (and how) changes or disruptions in the microbiota contribute to the association between obesity and cancer risk has received little attention in the literature. Dysbiosis associated with obesity may result in physiological changes that increase the risk of cancer [48]. Numerous investigations have demonstrated that certain bacterial taxa associated with obesity may contribute to the pathogenesis of CRC [79,80,81,82] (Table 1). There is currently no major universal obesity-related intestinal microbiota profile linked to the development of CRC because of the interpersonal variability that drives CRC, including genetic factors, behavioral characteristics, and diet. The CRC “driver-passenger” model posits that symbiotic “driver” bacteria initiate tissue malignancy through cellular DNA damage, and colorectal tumorigenesis is then facilitated by transformations in the intestinal microenvironment that give an advantage to “passenger” opportunistic pathogens such as Fusobacterium spp., Roseburia spp., and Streptococcus bovis [83]. In terms of a particular group of colonizing gut bacteria, it has been found that resident bacteria are essential for maintaining gut homeostasis. Patients with CRC have been observed to possess an altered quantitative relationship between the bacterial composition of the lumen and mucosa, characterized by a decreased rate of colonization of Faecalibacterium, Bifidobacterium, and Blautia, and a prevalence of Mogibacterium, Porphyromonas spp., and mucin-degrading species [84]. Moreover, the bacterium Clostridium bolteae, associated with inflammation, dyslipidemia, and insulin resistance, has been linked to the presence of oncogenic human polyomaviruses. The most commonly observed oncogenic viruses in the guts of both obese and cancer patients were BK polyomavirus and Merkel cell polyomavirus, implying that an obese microbiome could provide an opportunity for tumor-driving/driver bacteria and viruses to bring about cell transformation [85].

**Figure 1:**
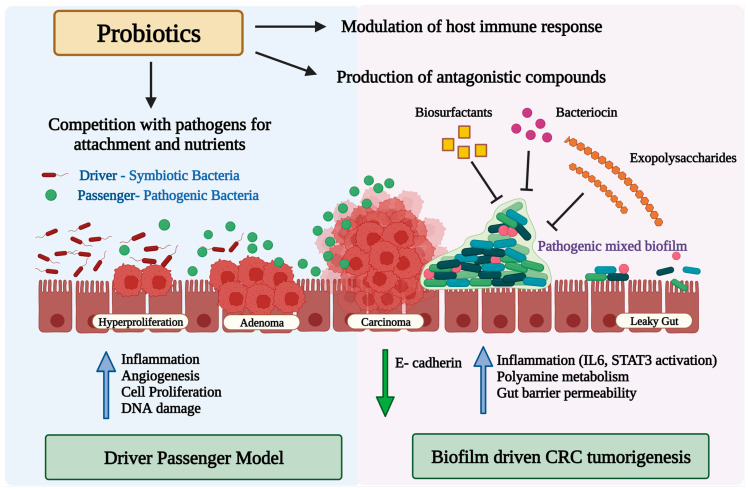
Diagram illustrating the potential role of the gut microbiota and microbial biofilm in colorectal cancer carcinogenesis, and the potential mode of action of probiotics against pathogenic bacteria alone or in biofilms. Upward blue arrows and downward green arrows show upregulation and downregulation, respectively.

Bacteria can contribute to carcinogenesis in multiple ways. Fusobacterium nucleatum has been shown to promote CRC progression through miRNA-mediated stimulation of Toll-like receptor (TLR)2 / TLR4 signaling and the suppression of apoptosis [86]. Peptostreptococcus produces metabolites that increase acid production, create a hypoxic tumor microenvironment, and promote bacterial colonization, all of which have pro-carcinogenic effects. Genotoxic bacteria including Fusobacterium nucleatum, Bacteroides fragilis, and certain strains of Escherichia coli carrying the polyketide synthase genomic island produce toxins such as cytolethal distending toxin, colibactin, hydroxyl radicals, or typhoid toxin, and induce DNA damage in IECs, which may initiate CRC development [87,88,89,90]. More recently, it has been demonstrated that these microbes instigate the development of CRC by forming biofilms. A polymicrobial community-based gut microbial biofilm development takes place in the inner layer of the colon [91]. Redistribution of colonic epithelial cell E-cadherin, increased gut epithelial permeability, impaired intestinal barrier function, elevated IL-6 production, and STAT3 activation are all caused by biofilm [91] (Figure 1). Along with the enhanced polyamine metabolism in colonic tissues, these microbial biofilms promote an inflammatory and pro-oncogenic condition that results in dysbiosis, onco-transformation, and the growth of tumors [91,92]. Secretion of secretory proteins known as secretomes or metabolites known as metabolomes make some bacteria carcinogenic via inducing the interactions between receptors in the host immune system and cancer cells [87,88]. The secretome is now referred to as the entire collection of proteins that are secreted into the extracellular space by a cell, tissue, organ, or organism at any given time and under any given set of circumstances via known and unknown secretory mechanisms involving constitutive and regulated secretory organelles [93]. The secretome encompasses functionally varied classes of molecules such as cytokines, chemokines, hormones, digestive enzymes, antibodies, extracellular proteinases, morphogens, toxins, and antimicrobial peptides, and can account for up to 30% of an organism’s proteome [94]. Several of these proteins are involved in a wide range of important biological activities, including cell adhesion, cell migration, cell–cell communication, differentiation, proliferation, morphogenesis, survival and defense, bacterial virulence factors, and immunological responses [94]. Proteins secreted by pathogens, in particular, mediate interactions with the host as these are present or active at the interface between the pathogen and the host cells [95]. Metabolomes are made up of several metabolic by-products of gut microbiota metabolism and oncometabolites implicated in carcinogenesis. Tumor metabolites such as succinate, lactate, D-2- or L-2-hydroxyglutarate, and fumarate accumulate in the cancerous cells after their metabolism. Furthermore, some metabolites generated by the microbiota, such as butyrate, can inhibit pro-inflammatory genes and tumor growth, while lactic acid can provide fuel for cancer cells and encourage their spread. Gut microbiota dysbiosis can contribute to the development and progression of CRC due to the wide-reaching implications of this imbalance, such as the generation of a chronic inflammatory state or immunological response, alterations to stem cell dynamics [96,97], the production of toxic and genotoxic chemicals, and the disruption of host metabolism.

After CRC surgery, recurrence incidence can be as high as 40% during the first three years, with local recurrence happening in 1–23% of cases [98]. Several studies have suggested a link between obesity, gut dysbiosis, and CRC recurrence after resection [99,100,101,102], wherein chronic inflammation is believed to play a role in this link. Obesity is associated with chronic low-grade inflammation, which can lead to alterations in the gut microbiota. Additionally, anastomotic leakage (AL) and surgical site infections could result from the altered postoperative composition of the intestinal microbiota, which could also increase the virulence and number of pathogens and decrease the number of beneficial microorganisms [101]. These alterations can then lead to an increased risk of CRC recurrence after resection. Collagenolytic bacteria such as *E. faecalis* and *P. aeruginosa,* which comprise the prominent examples of this pathobiome, particularly in AL [103], have the ability to cause inflammation by activating MMP-9. There is evidence that patients with CRC who have high MMP-9 levels have tumor invasion and poorer prognosis [103]. High *F. nucleatum* levels were linked to CRC recurrence, possibly due to chemotherapy resistance from autophagy pathway stimulation [104]. Further research is needed to fully understand the mechanisms underlying relationships and to develop effective interventions to prevent/reduce the risk of CRC recurrence in obese individuals with gut dysbiosis.

## 4. Obesity-Associated Alterations in the Gut Microbiome That May Instigate CRC 

**Figure 2:**
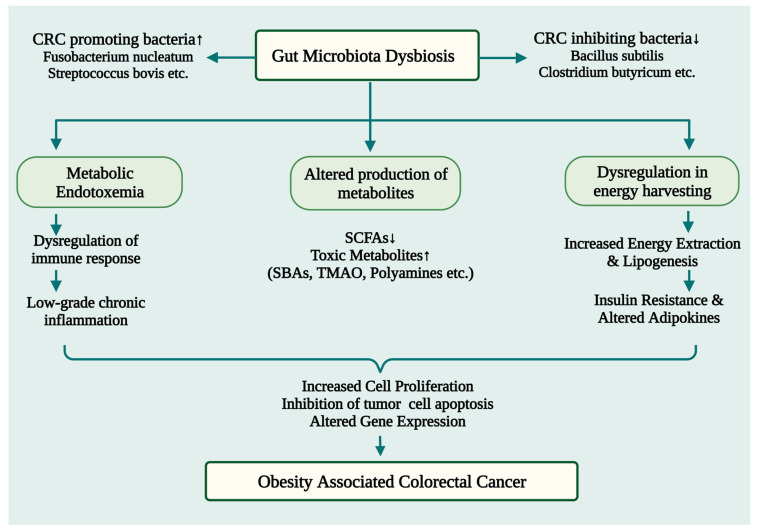
A schematic illustration depicting the potential ways by which obesity-induced gut microbiome dysbiosis may lead to CRC development. SBAs: Secondary Bile Acids; TMAO: trimethylamine-N-oxide; SCFAs: Short chain fatty acids; CRC: Colorectal Cancer. ↑ Upregulation; ↓ Downregulation.

CRC is triggered by a dysbiosis of the gut microbiota induced by obesity in several ways. The main processes are as follows: (1) intestinal microbiota dysbiosis can cause low-grade chronic colonic inflammation, which promotes colorectal tumorigenesis; (2) intestinal microbial metabolism in the obese state can contribute to the formation of toxic metabolites that can be carcinogenic; (3) disturbed gut microbiota can increase energy harnessing and nutrient availability, leading to metabolic disturbances (e.g., insulin resistance and altered adipokine function) that promote tumor growth (Figure 2).

### 4.1. Obesity and CRC: Is Gut Dysbiosis-Induced Inflammation the Missing Link?

Inflammation is the physiological homeostatic response of the body’s innate system to infectious and non-infectious pathogens that alters vascular and immunological cell activity. The immune response to pathogens can lead to inflammation, which is a normal part of the resolution process. However, inflammation may contribute to the growth and spread of tumors if it continues unabated.

Intestinal inflammation and DNA damage of the intestinal cells due to the introduction of genotoxins have been suggested as the potential mechanisms linking gut microbial dysbiosis with carcinogenesis [105]. Obesity and CRC pathogenesis are both characterized by chronic low-grade inflammation [106]. Dysbiosis upsets the balance between pro- and anti-inflammatory cytokines, affecting intestinal homeostasis and accelerating the disease’s progression. Obesity and IBD-related chronic inflammation are risk factors for CRC. Inflammation has the potential to damage DNA and eventually lead to the development of cancer by producing reactive oxygen and nitrogen species that cause oxidative stress and increase cell turnover. Both sporadic and colitis-associated CRC is known to be largely influenced by inflammation and inflammatory cytokines [107]. HFD-fed mice have increased colonic TNF-α expression, which is a powerful inducer of IL-6 that is known to contribute to the promotion of CRC [108]. Colonic inflammation, which is linked to the development of CRC, has been demonstrated in numerous studies to be significantly influenced by intestinal microbiota [109,110,111] (Table 1). Furthermore, modifications in the intestinal barrier’s permeability brought on by obesity may also have an impact on the development of CRC. The combination of pre-existing adipose tissue inflammation and metabolic endotoxemia, which results from obesity-related gut barrier dysfunction, increases systemic inflammation, which encourages tumor growth and aids in the production of pro-inflammatory cytokines. The hypothesis that dietary dysbiosis alone is sufficient to promote inflammation is further supported by the fact that the transfer of gut microbiota from HFD mice to germ-free mice increased the stimulation of the NfkB1 inflammatory pathway [112]. These studies suggest that inflammation due to obesity-mediated intestinal dysbiosis and intestinal barrier dysfunction play a role in the increased risk of CRC in obese subjects. Therefore, it is important to consider the potential contribution of these factors to effectively reduce the risk of CRC in obese individuals.

Our increasing comprehension that changes in the microbiota precede obesity and the findings that altered microbiota-pattern recognition receptor (PRR) communications promote inflammation implicate inflammation as a possible key mechanism by which the microbiota modulates the association between cancer and obesity. Numerous studies have shown that eating foods high in saturated fat affects the quality and load of the microbiota, which negatively affects GI health [113,114]. Along with increased hyperplasia of visceral fat, epithelial permeability, and endotoxemia, animals that are predisposed to developing insulin resistance in response to a HFD also have altered microbiota (for example, decreased abundance of *Enterococcus* spp., *Clostridium leptum*, and *Nitrospira* spp. [115]. While permitting some nutrients and electrolytes to penetrate through, the intestinal epithelia play a crucial part in preserving barrier integrity. Numerous studies utilizing both murine and human models have linked obesity to higher endotoxemia, which undermined the epithelial barrier and increased luminal LPS permeability because tight junction protein (ZO-1) expression was downregulated [116,117,118,119,120]. In animal models, intestinal barrier leakage elevated endotoxemia, and stimulation of pro-inflammatory IL-23/IL-17 signaling that promotes tumor growth are all associated with the development of CRC [121,122,123]. The gut’s luminal LPS absorption is assumed to be caused by either LPS leakage via the gut epithelia’s tight junctions or LPS absorption via lipid-rich chylomicrons. These pathways can cooperate to generate metabolic endotoxemia and are not mutually exclusive [124]. The gut barrier works as a complex system of interconnected components to prevent the gut microbiota from colonizing epithelial cells and to limit the entry of pathogen-associated molecular patterns (PAMPs) such as LPS [125]. Obesity-induced microbial imbalances in the gut can be caused by a thinner mucous membrane, uneven distribution and localization of tight junction proteins (TJP), and an aberrant immune response involving immunoglobulin A (IgA) and antimicrobial peptides. These defects combine to allow LPS leakage, which then triggers the TLR4 myeloid differentiation primary response protein (MYD88) reaction and NF-κB, culminating in the generation of an inflammatory response [37,125,126]. Leaky gut, increased levels of the pro-inflammatory cytokine IL-1β, and the harmful bacterial metabolite trimethylamine-N-oxide (TMAO) have been seen in OB-CRC patients, which may be related to their unique intestinal microbial profile marked by an excess of opportunistic pathogens and insufficiency in butyrate-producing bacteria [48]. Inflammatory signals can activate several kinases, including c-Jun N-terminal kinases (JNK) and IkappaB kinase (IKK), that regulate transcription factors, most notably NF-κB, and inhibit insulin action. Interaction between LPS and TLR4 activates a downstream signal cascade that causes Iκβ to become phosphorylated, which then leads to the relocation of NF-κB and initiates the transcription of proinflammatory genes. Inflammation can influence many of the key molecular and cellular processes needed for tumorigenesis, such as the interplay between external and internal signals, which can affect genomic instability, cell proliferation, survival, and abnormal differentiation. An example of this is the activation of the STAT family members (especially STAT3) that often occurs in tumor development in different tissues and links to CRC’s oncogenic process [127]. STAT3 boosts the expression of anti-apoptotic genes, which leads to cell growth and survival by promoting cyclin D family members and the proto-oncogene Myc. Many studies have shown that inflammatory processes activate the NK-κB-IL-6-STAT3 signaling cascade, resulting in initiating or advancing of cancer. Therefore, the changes to the microbiota associated with obesity may cause cancer progression by activating these inflammatory pathways [127]. 

### 4.2. Gut Microbial Metabolites as Key Regulators of Obesity and Related CRC

The link between obesity and cancer is complex and the precise mechanism by which the gut microbiota may be involved is not yet fully understood. However, it is known that the microbiota can affect nutrient metabolism and the production of metabolites that may raise cancer risk. Specifically, the gut microbiota can transform dietary environmental toxins and chemicals into obesogenic and diabetogenic substances, which can contribute to the development of gastrointestinal tumors. 

Additionally, the microbiota produces a wide range of metabolites from ingested food and host metabolic products, including SCFAs and SBAs, and some of these compounds may become toxic or carcinogenic when exposed to certain physiological conditions. Research is ongoing to better understand the role of gut microbiota in the development of cancer and the prevention of obesity-related cancers.

The gut microbiome plays an important role in the health of the digestive system and in the prevention of CRC. Gut bacteria, such as *Clostridium cluster IV* and *Faecalibacterium,* produce tumor suppressor metabolites, such as SCFAs, through the fermentation of dietary fiber [128]. SCFAs, such as butyrate, acetate, propionate, and valerate, regulate immune responses and help to maintain a healthy immune system. SCFAs can also enhance intestinal barrier performance by promoting connexin expression and mucus formation, as well as reducing the amount of oxygen in the intestinal cavity. Consequently, SCFAs are effective in preventing the development of CRC [109,128,129] (Table 1). Colon cancer is a serious disease that can have a wide range of effects on the body. Recent research has demonstrated potential anti-carcinogenic properties in cellular and animal models when exposed to SCFAs [130,131,132,133]. Notably, butyrate, acetate, and propionate all have positive effects on health, with butyrate being the most effective. Butyrate has anti-inflammatory and anticancer properties, which are believed to be mediated through cell metabolism, microbiota homeostasis, immunological control, and gene epigenetic modification [128]. In particular, butyrate serves as the primary source of energy for colonocytes and controls epithelial proliferation. Furthermore, it prevents histone deacetylase activity in colonocytes and immune cells, which reduces the production of pro-inflammatory cytokines and causes CRC cells to undergo apoptosis [134]. SCFAs can also significantly lower fecal pH in the colon, thus inhibiting pathogen growth and DNA damage, promoting apoptosis, and limiting cancer cell proliferation. Furthermore, butyrate and propionate shape the mucosal immune system by controlling the differentiation of colonic regulatory T cells and suppressing colonic inflammation and carcinogenesis [109,129,135]. Overall, SCFAs have the potential to be a powerful tool in the fight against cancer, with butyrate being the primary one. 

An HFD- or obesity-related modification in the gut microbiota results in a decrease in bacteria that produce SCFAs, mostly butyrate-producing bacteria, and an elevation in pathogenic bacteria, which affects the synthesis and absorption of SCFAs. Pathobiont overgrowth impairs barrier function, induces inflammation, and downregulates the receptor and transporter for SCFAs, as seen in IBD patients and in vivo IBD models [136,137,138,139]. A major cause of CRC is the chronic inflammatory condition associated with IBD. Due to this, individuals with advanced colorectal adenoma have a higher chance of developing CRC when their SCFA levels are lowered [140]. According to clinical case studies, there were lower levels of fecal SCFAs in CRC patients compared to control groups. This is likely due to fewer SCFAs-producing organisms such as *Lachnospiraceae, Roseburia* spp., and *Bifidobacterium* spp. [52,141]. Furthermore, compared to nearby mucosa, metabolomic investigations have shown considerable abnormalities of SCFA metabolism in CRC [142]. SCFAs have been shown to inhibit colitis and the accompanying carcinogenesis by binding to the receptor Gpr109a [135]. Activation of this receptor has been demonstrated to cause an increase in IL-18 production in intestinal epithelial cells, which in turn can stimulate the repair of mucosal tissue. This is done by controlling the production and availability of IL-22 [143]. In murine models, the absence of IL-18 has been linked to dysbiosis of the gut microbiota, a disruption of the inflammatory response, and a disruption of homeostasis and mucosal repair [144,145], all of which can lead to an increased risk of CRC carcinogenesis [143,146,147].

Given the importance of SCFAs, numerous epidemiological studies have revealed that people with diets low in SCFAs or low fecal SCFA levels have an increased risk of developing inflammatory diseases and cancer, particularly breast and gastrointestinal cancers [148,149]. It is now believed that high-fiber diets can have anti-cancer effects due to the formation of SCFAs such as butyrate through the action of the microbiota [150]. Although SCFAs, can shield the host from diet-induced obesity, excessive SCFAs may provide extra energy to the host that may promote obesity. Thus, SCFAs might act as a double-edged sword and this aspect needs to be further explored.

Recent research has suggested that bacteria, including *Fusobacterium nucleaturm*, *Helicobacter pylori*, *Streptococcus bovis*, *Clostridium septicum*, and *Bacteroides fragilis*, are involved in the carcinogenesis of CRC [151]. The gut microbiota can contribute to carcinogenesis in a variety of ways. Risk factors such as host genetics, obesity, and an HFD can lead to an alteration of the gut microbiota, resulting in reduced mucus layer thickness, high intestinal permeability, and the translocation of commensal microbiota and its metabolites. Maleficent bacteria can produce large amounts of pathogen-associated molecular patterns (PAMPs) such as LPS that can be recognized by the TLR4 of macrophages and dendritic cells, which then release pro-inflammatory cytokines (IL-1β, TNF-α, IL-23). Other unfavorable metabolites produced by the gut microbiota, such as SBAs, hydrogen sulfide (H2S), TMAO, and N-nitroso compounds (NOCs), may also change the ecological makeup and metabolic activity of intestinal microorganisms, leading to an increase in sensitivity to carcinogenic stimuli. These metabolites may lead to DNA damage and low-grade inflammation, activate tumorigenic signaling pathways, and control tumor immunity, leading to an increased risk of CRC [152]. Furthermore, these metabolites may directly affect IECs after crossing the mucosal barrier, induce immunological responses in the intestinal stroma, release pro-inflammatory signals such as TNF and IL-17, or cause immunosuppression in the tumor microenvironment (TME). NOCs may also contribute to DNA damage through DNA adducts and DNA alkylation, as well as increased production of ROS [152].

Obesity or an HFD may cause the liver to produce more bile acids, which in turn causes more bile acid to accumulate in the gut. The gut microbiota produces 7-dehydroxylase, which turns primary bile acids into SBAs. SBAs are mostly composed of deoxycholic acid (DCA), lithocholic acid (LCA), and a trace quantity of ursodeoxycholic acid (UDCA), and levels of these acids have been linked to CRC, particularly DCA, which is thought to be a carcinogen [153]. The development and progression of CRC is a multistep, multigene, multi-pathway process involving gene mutations, DNA mismatch repair gene inactivation, chromosomal instability, and multiple abnormalities in cell signaling pathways that is influenced by a variety of factors [153]. SBAs, especially DCA, are considered to be genotoxic because they can cause DNA damage in intestinal mucosal epithelial cells [128] and facilitate intestinal tumorigenesis and progression [154]. DCA induces genomic instability through several mechanisms, including mitochondrial and endoplasmic reticulum damage, oxidative DNA damage, chromosomal aneuploid mutations, and increased micronuclei [155]. Additionally, it suppresses CRC cell apoptosis while encouraging CRC cell proliferation [156,157]. To stimulate CRC growth, SBAs also interact with a number of intracellular transduction networks, including the PKC-p38 MAPK signaling pathway, the EGFR-ERK1/2 signaling system, and the Wnt/β-catenin signaling circuit [153]. Furthermore, the propensity of bile acids to dissolve the phospholipid bilayer to provide antimicrobial qualities results in alterations to the gut flora and an increase in pathogens. Therefore, an HFD can enhance the level of SBAs in CRC patients, which can further affect colonic epithelial cell proliferation and apoptosis, erode the gut barrier, modify the intestinal microbiota, and promote the occurrence of CRC [153]. Therefore, it is essential to be aware of the potential risks associated with the presence of these molecules to reduce the risk of CRC.

The above-mentioned well-known compounds as well as lesser-known ones including polyamine, ammonia, heterocyclic amines (HCAs), and lactate are all examples of the varied reservoir of metabolites from intestinal microbiota that play a role in the development and promotion of CRC [152]. When meat is cooked at a high temperature, a family of mutagenic substances known as HCAs is generated by the reaction of creatine (or creatinine), amino acids, and sugars. The HCAs are transformed into genotoxic metabolites after being consumed and absorbed [158,159]. Numerous studies have postulated HCA as an oncogenic agent for CRC due to the correlation between greater HCA levels and a higher risk of CRC [160,161,162]. According to reports, HCAs can cause oxidative base damage, strand breakage, microsatellite instability, frameshift mutations, and microsatellite instability [163]. More specifically, mutations in the colon can be brought on by HCA-induced DNA adducts, which are regarded to be the main executor of DNA-damaging and carcinogenic properties [164].

Products of protein fermentation such as polyamines and ammonia are among the gut microbiota-derived compounds having cancer-promoting effects [165]. To fulfil their enormous metabolic needs, most cancers have a significantly elevated need for polyamines as they are essential for cell growth and differentiation [166]. Through strictly controlled production, breakdown, absorption, and output routes, intracellular polyamine levels are kept at a constant level. Interestingly, a recent metagenomic study discovered a connection between the altered polyamine metabolism and the microbiota linked to CRC, indicating that these metabolites may be crucial to the onset and progression of CRC [167]. Colonocytes that are continually exposed to free ammonia may play a role in the development of CRC [152]. Serum ammonia levels are elevated in CRC patients [168]. High ammonia concentrations cause T cells to undergo metabolic reprogramming, which reduces proliferation and enhances exhaustion. The ammonia-related gene profile correlates with aberrant T-cell responses, poor patient prognosis, and inability to respond to immunotherapy. Ammonia removal leads to the reactivation of T cells and lowers the risk of CRC [168].

Another metabolite, lactate, can serve an immunosuppressive effect in the TME and promote tumor formation by attracting and stimulating the function of immunosuppressive molecules and cells [169]. It has been found to boost angiogenesis and provide oxygen and glucose to cancer cells, which in turn facilitates the growth, invasion, and migration of colon cells [169]. The lactate metabolized by cancerous cells exacerbates CRC progression by prompting macrophages to exhibit M2-type polarization and the production of high-mobility group box 1 (HMGB1), which is essential in maintaining nucleosome structure and controlling the transcription of many genes [170]. In addition, the accumulation of lactate in the TME creates an acidic environment that impairs the cytotoxic and effector capabilities of T cells, thus enabling tumors to escape the immune system and reducing the effectiveness of certain chemotherapy drugs, all of which have a detrimental influence on CRC prognosis [152]. 

Although the pathogenic effects of numerous gut microbiota metabolites on CRC have been widely reported, their precise mechanisms are still not fully understood. In addition, there is an ongoing debate regarding the dual nature of some metabolites, as they have both anti-carcinogenic and pro-tumorigenic properties. These properties may vary depending on the concentration of the metabolites in the lumen, interactions with certain other metabolites, the length of colonic stasis, and the stage of tumor development. To gain a better understanding of the role of metabolites in CRC progression, further research is necessary.

### 4.3. Obesity-Induced Dysregulation in Energy Homeostasis Metabolism Links Gut Microbiome and CRC

A lifestyle that is related to energy imbalance, excess body fat, a poor diet, inactivity, and sedentary behavior are risk factors for CRC. These factors are particularly prone to clustering and share similar pathways in colon carcinogenesis, such as chronic systemic inflammation and insulin resistance.

Within the ecosystems of human bodies, cancer cells and microbes coevolve, and both are dependent on external nutrients for life and reproduction. This implies that our diet—and specifically, whether we have a surplus of energy and nutrients—can influence how quickly both cancerous and microbial cells develop. Furthermore, the multiplication and survival of cancer cells and microorganisms can be influenced by one another through the synthesis of certain metabolites. Under ideal circumstances, the growth and proliferation of microbes and the host’s somatic cells are limited by the availability of resources such as protein, carbohydrates, and fat, as well as by somatic cell cycle regulators. Most human cells prefer glucose as a fuel source, and from this source, cellular energy in the form of ATP is used to power cellular operations. This oxygen-dependent route shifts to less effective mechanisms such as fermentation in the event of cancer. This metabolic malfunction, also known as Warburg metabolism, produces lactate, which damages the extracellular environment and promotes metastasis and invasion into new tissues [171]. The breakdown of xenobiotics, such as drugs and environmental pollutants, digestion of complex carbohydrates, and the synthesis of necessary amino acids and beneficial fatty acids are all key aspects of human metabolism that are influenced by interactions between bacteria and human cells. As cancer progresses, these metabolic connections between bacteria and human cells may change from ones that promote health to ones that harm it as microbes start interacting with cancerous cells instead of healthy human cells. It has been discovered that microbial dysbiosis aids in the development of gastrointestinal cancer. In addition, it is believed that this dysregulated energy homeostasis and risk of CRC are related to the metabolic effects of obesity. Obese people tend to have a higher calorie intake, which can cause an elevation in the circulating fatty acid and glucose levels in the body. This can further upset the delicate balance of energy homeostasis and possibly lead to additional disturbance of the gut microbiota. Gut microbial metabolism may act as a mediator in this relationship between excessive energy consumption and CRC. Studies on the effects of caloric restriction imply that improved cancer outcomes result from increased gut microbial diversity and a consequent decrease in inflammation [172,173,174]. Improved gut barrier integrity, which reduces the translocation of microbially derived inflammatory markers such as LPS, as well as advantageous shifts in microbial abundance (such as an increase in *Lachnospiraceae* abundance), which have been seen in obese women on very low-calorie diets (800 kcal/day), may be the mechanisms underlying this [172]. The gut microbiota may control energy storage by supplying lipogenic substrates (monosaccharides, SCFAs) to the liver and upregulating the activity of the lipoprotein lipase (LPL) enzyme as a result of suppressing the fasting-induced adipose factor in the gut [175]. 

These results imply that gut microbial dysbiosis may promote energy harnessing or disrupt the storage of energy in the host by altering various metabolic hormones, such as insulin, adiponectin, and leptin, thereby resulting in an unfavorable energy balance and predisposition to obesity [175]. Alterations in energy balance-responsive hormones could increase the likelihood of CRC and other cancers. Numerous pro-tumorigenic effects of insulin resistance include increased levels of systemic TNFα, increased NF-κΒ activation, activation of the mTOR pathway, and increased proliferative/survival signals mediated by IGF-1 [176]. Obesity-induced dysregulation in energy homeostasis metabolism, which links gut microbiota to CRC, is a significant topic of research. The processes underlying this correlation are currently being researched; nevertheless, knowing the mechanisms that underpin this connection could provide insights into potential therapeutic approaches. Dietary therapies, for example, aimed at restoring energy homeostasis balance, may be effective in lowering the risk of developing CRC.

## 5. Potential Applications of the Gut Microbiome in Clinical Practice

The gut microbiome has a variety of possible roles in the management of CRC; for instance, it may be used as a biomarker for screening, prognosis, and/or prediction, as well as a modifiable element influencing the efficacy of systemic CRC treatment. Due to their greater prevalence in fecal samples from patients with adenomas and CRC, certain bacterial species, such as *F. nucleatum*, can be employed as screening markers [177,178]. Other screening markers, such as the metabolic metabolites and genotoxic byproducts of some strains, may be used to identify and screen CRC in its initial stages. A patient’s clinical outcome, responsiveness to treatment, and potential adverse effects may all be predicted by the gut microbiota, which can also function as a predictive and/or prognostic biomarker. For instance, in a study, larger levels of *F. nucleatum* in CRC tissue were linked to worse clinical outcomes, such as shorter survival periods and a poorer prognosis [179]. Even though only a few studies using various metabolomic approaches have demonstrated the diagnostic value of metabolites such SCFAs [180,181], feces metabolome screening may be another possible non-invasive tool for creating a specific metabolic fingerprint to identify CRC [182]. The notion that metabolic changes can be used as indicators of an early precancerous environment in screening processes has not been tested and verified on a large scale yet, but this could be a valuable approach.

### 5.1. Modulation of Gut Microbiome

**Figure 3:**
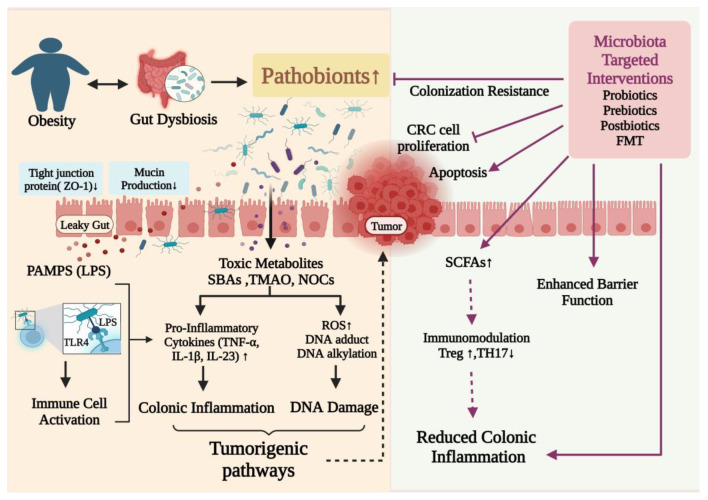
Putative mechanisms of action of gut microbiome–targeted therapeutic interventions in the treatment of obesity and associated CRC. PAMPS: Pathogen-associated molecular patterns; LPS: lipopolysaccharides; ROS: reactive oxygen species; TNFα: tumor necrosis factor alpha; SBAs: secondary bile acids; TMAO: trimethylamine-N-oxide; NOCs: N-nitroso compounds. ↑ Upregulation; ↓ Downregulation.

One of the primary processes behind the association between obesity and cancer is gut microbial dysbiosis, and effective strategies to repair the gut microbiota potentially present novel treatments that may reduce the risk of CRC associated with obesity. By modifying the gut flora in high-risk individuals, CRC can be prevented, and chemotherapy and immunotherapy treatments can be more effective. Probiotics, prebiotics, postbiotics, FMT, and bacteriophage therapy are all methods for positively modifying the gut microbiome. These biotherapies balance bacterial populations and encourage their beneficial metabolic activity to create a healthy gut environment (Figure 3).

#### 5.1.1. Probiotics

Probiotics are microbes, including bacteria, yeasts, and molds, that, when administered in adequate quantities, can improve the host’s health. The most popular bacterial genera utilized as probiotics include *Lactobacillus*, *Bifidobacterium*, *Bacillus, Streptococcus*, and *Enterococcus* [183]. Probiotics may have an impact on the gut in the prevention and treatment of CRC through mechanisms such as immunomodulation by promoting homeostatic immune responses, supporting the expansion of Treg cell-mediated anti-inflammatory responses, modulating the release of pro-inflammatory cytokines, supporting production of anticarcinogenic and antimicrobial compounds, enhancing the host’s antioxidant system, degrading carcinogens, inhibiting the colonization of pathogenic bacteria, improving the function of the gut barrier by upregulating the synthesis of mucin and tight junction proteins, and encouraging apoptosis in CRC cells [184].

The administration of probiotics has a strong preventive effect against CRC, according to several investigations using chemically-induced animal models. *Faecalibacterium prausnitzii*, a putative probiotic, inhibits the NF-κB pathway in intestinal epithelial cells and prevents colitis in animal models by producing hydrophobic microbial anti-inflammatory compounds [185]. Treatment with a probiotic combination of *L. acidophilus*, *L. plantarum*, and *Bifidobacterium longum* enhanced the number of cell junction proteins, enhancing the integrity of the intestinal mucosal barrier in CRC patients [186]. Patients who had undergone resection experienced less colorectal tumor atypia while taking the probiotic orally [187]. Single-genus and multi-strain probiotics (*L. fermentum* NCIMB 5221 and *L. acidophilus* ATCC 314 in vitro and in vivo study) [188], single-strain probiotics (L. acidophilus or *L. rhamnosus* GG; *L. plantarum*; *L. casei* BL23 in vivo study) [189,190,191] and multigenus and multistrain probiotics (VSL#3 containing, *B. longum*, *B. breve, B. infantis*, *L. casei*, *L. acidophilus*, *L. plantarum*, *L. bulgaricus*, and *S. thermophilus* along with balsalazide in vivo study) [192] are some of the probiotic strains that have been described as adjuvant treatment agents for CRC management. Intervention of *B. lactis* and *L. acidophilus* patients with CRC showed higher levels of butyrate-producing bacteria such as *Clostridiales* spp. and *Faecalibacterium* and lower levels of CRC-associated taxa including *Peptostreptococcus* and *Fusobacterium* [193]. Diverse probiotic supplements have been demonstrated to considerably minimize postoperative complications, improve postoperative quality of life in CRC patients [194,195,196,197] and gastrointestinal function, and reduce the frequency of diarrhea [198]. In addition, permeability of horseradish peroxidase, lactulose-mannitol ratio, bacterial translocation, enteropathogenic bacterial load, and infection incidence were all significantly decreased, while tight junction protein expression, transepithelial resistance, and serum zonulin levels were all improved in CRC patients on taking probiotics [186,199]. Probiotics can also work through a variety of methods to inhibit biofilm formation and biofilm pathogen survival. Among these processes are competition with pathogens, the manipulation of host immunological responses, and the synthesis of antagonistic substances such as exopolysaccharides, bacteriocins, and biosurfactants that have antibiofilm action [91,200,201]. These antagonistic substances have been demonstrated to hinder the adhesion and development of biofilms as well as the thinning of mature biofilms.

The positive effects of probiotic supplementation depend on the host’s physiology, the severity of the condition, the strain, the dosage, the length of the intervention, other dietary supplements, etc. Probiotic supplements strengthened the immune system and intestinal health in CRC patients, boosted antimicrobial defense, and neutralized carcinogenic chemicals. However, not all probiotic treatments have noticeable advantages for CRC patients’ health. To enhance the functionality of enhancing therapeutic efficacy, the dosage of probiotics provided to patients in the future should be evaluated through preclinical and clinical experiments. To determine the precise mechanism and the potential of probiotics in CRC prevention, more research is highly advised.

#### 5.1.2. Prebiotics

Prebiotics are selectively fermented substances that elicit specific changes in the makeup and/or activities of the host’s gut microbiota. The polyunsaturated fatty acids (PUFAs), polyphenols, and carbohydrates such as inulin, fructans, fructooligosaccharides (FOS), galactooligosaccharides (GOS), and xylooligosaccharides (XOS) possess prebiotic properties. Prebiotics function through a variety of mechanisms, including selective induction of the growth or activity of beneficial intestinal bacteria, fermentation via gut microbiota, direct uptake by the intestine and anti-inflammatory activity, and preventing pathogen colonization by interacting with them.

Numerous studies in animal models have demonstrated that supplementing different amounts of FOS and GOS to diets may alter the composition of gut microbes and encourage the growth of advantageous bacteria such as *Lactobacillus, Bifidobacterium, Akkermansia, Ruminococcus, Roseburia*, and *Faecalibacterium* [202,203,204]. This microbiota regulation can considerably reduce body weight and total fat while improving gut barrier performance [33]. Furthermore, better gut barrier integrity improves glucose tolerance and insulin resistance in mouse models [205]. Recent studies on animal models that were genetically predisposed to CRC or were exposed to induction carcinogens such as azoxymethane/dextran sodium sulfate (AOM/DSS) found that different prebiotics, including GOS derived from lactulose, inulin, and phenolic compounds such as anthocyanins and ellagic acid, had inhibitory effects on the development of CRC. Notably, the use of FOS allows researchers to observe the beneficial effects of prebiotics on the development of CRC in a variety of human colon cell lines [206]. Zheng et al. [207] formulated spores-dex, i.e., prebiotics-encapsulated probiotic spores containing *C. butyricum* (as a probiotic) and chemically modified dextran (as a prebiotic) and evaluated their anti-cancer effects in colon tumor models [207]. As a result of *C. butyricum* fermenting dextran inside the lesions, SCFAs with anti-cancer potential were produced. Additionally, they also boost the number of SCFA-producing bacteria, such as *Eubacterium* and *Roseburia*, considerably slowing the growth of tumors. SCFA-producing bacteria help to inhibit tumors by creating a tumor-suppressing microenvironment in the gut [207]. Furthermore, SCFAs have been shown to induce CRC apoptosis, modulate oxidative stress, improve the epithelial barrier, and suppress inflammation. The novel combination of prebiotics GOS and inulin exhibited stronger preventive activity against CRC by inhibiting aberrant crypt foci formation and biomarkers of colon cancer in the murine models [208]. Prebiotics can be added to the diet to regulate and enrich the intestinal microbiota, with a focus on biologically active substances found in foods of plant origin that can prevent or attenuate the development of CRC.

#### 5.1.3. Postbiotics

Postbiotics are bioactive compounds produced during fermentation carried out by probiotic live cells in the intestine and offer various health benefits to the host. These may include inactivated microbial cells, microbial cell fractions, cell metabolites, functional proteins, and others. Considering that postbiotics are found in the conditioned/supernatants media of bacterial culture, they are comparatively safer than live microbes. They function as anti-tumor agents by specifically suppressing tumor cells while protecting the intestinal epithelium by preventing apoptosis of epithelial cells and boosting IgA production. In colon cancer cells, *Saccharomyces boulardii*-derived postbiotics reduced cell viability, inhibited the first (G0/G1) phase of cell division, affected the nucleus of the treated cells, and induced apoptosis by decreasing the expression of RelA and Bcl-XL genes and elevating the expression of Caspas3 and PTEN genes [209]. Postbiotics modify the makeup of the gut microbiota and the function of the immune system, in addition to enhancing the efficacy of CRC treatment and minimizing its side effects in CRC patients because of their anti-inflammatory, anti-proliferative, anti-oxidant, and anti-cancer capabilities [209,210]. Postbiotics can reportedly be used as promising drugs for both preventative and adjuvant therapy strategies in CRC patients without having any noticeable negative side effects. This is owing to their particular economic (low production costs) clinical (safe origin), and technical (stability) qualities.

#### 5.1.4. Fecal Microbiota Transplantation (FMT)

FMT is a fascinating and revolutionary biotherapy that entails introducing the intestinal tract of a person with an illness to the fecal liquid from a healthy donor to recreate a healthy microbiome and manage a range of health problems. FMT has been approved for the treatment of *C. difficile* infection (CDI) [211] but has also shown promise in the treatment of obesity and associated disorders. It has been shown to restore microbial balance in unbalanced communities and can be used to manage multiple gastrointestinal ailments [212]. According to research by Rosshart et al., transplanting the feces of wild mice into laboratory mice can improve resistance to CRC induced by mutagen/inflammatory drugs while also promoting fitness [213]. Intriguingly, Wong et al. showed both conventional and germ-free mice fed the carcinogen azoxymethane produced more tumors and had lower microbial abundance when exposed to fecal microbiota from CRC patients [214]. Additionally, the animals increased the frequency of Th1 and Th17 cells and elevated C-X-C motif chemokine receptor 1 (CXCR1), CXCR2, IL-22, IL-17A, and IL-23 [214]. Metagenomic and metabolomics data indicate that FMT and anti-PD-1 therapy are synergistic in the treatment of CRC [215]. FMT increases the effectiveness of anti-cancer therapy, which may aid in the development of novel microbiota-based anti-cancer treatments. While FMT has been successful, there is a lack of control as the entire gut microbiota is transferred with the therapeutic microorganisms. It is thus essential to be mindful of the donor’s health and the composition of their gut microbiome for optimum results. The success of this method depends on having clear inclusion and exclusion criteria for donors.

#### 5.1.5. Bacteriophage Therapy

Recent advances in scientific research have led to the discovery of various potential treatments for CRC. Bacteriophage therapy is among the most promising treatment options used for the modulation of gut microbiota and involves the use of viruses that infect and replicate in bacteria. This type of treatment has been shown to induce bacterial and metabolic changes in a gut microbiome model, as well as modulate the human immune system, suggesting it could have an important role in the treatment of disorders including obesity and cancer brought on by gut microbiota dysbiosis. The precise mechanism of action of phage therapy in cancer is not yet clear, but researchers believe it involves an immunological response triggered by the presence of the virus. In a co-culture system with HCT116 colon cancer cells, a recent study using the lytic bacteriophage EFA1 discovered that it could break *E. faecalis* biofilms and alter the growth-stimulatory effects of *E. faecalis* [216]. To fully comprehend phage therapy in cancer and its potential uses, more research on the bacteriophage EFA1 in these systems and in vivo models is suggested. A decrease in immunosuppressive myeloid-derived suppressor cells (MDSCs) at the tumor site has been linked to treatment with the bioinorganic hybrid bacteriophage M13@Ag. The host immune system may be stimulated, and CRC may be suppressed as a result of activating antigen-presenting cells. Additionally, studies have shown that M13@Ag in conjunction with either immunotherapy or chemotherapy may be able to prolong overall mice survival in an orthotopic CRC model [217]. Bacteriophages have the potential to provide significant benefits in cancer treatment, including their ability to kill carcinogenic bacteria, modulate the immune system, and introduce toxins to the TME. However, further study is needed to understand how exactly bacteriophages interact with tumor cells, bacteria, and the TME as a whole. This knowledge could help to optimize the use of bacteriophages to treat cancer and thus should be a priority in cancer research. Additionally, efforts must be made to ensure that the use of bacteriophages does not lead to the inadvertent introduction of new forms of bacteria that could have a deleterious effect on the TME. Overall, bacteriophage therapy seems like a viable alternative for treating CRC. The available data point to the therapy’s safety and efficacy, but additional study is required to fully grasp its potential and create more efficient therapies.

## 6. Conclusions and Prospects

It is crucial to comprehend the causes of CRC because the disease is widespread and has a high fatality rate. Through its impact on metabolic and immunological processes, the intestine’s microbial environment has a significant impact on human physiology. Disturbances in intestinal homeostasis and the gut microbiota brought on by obesity may favor the development of certain diseases, including CRC. Due to the strong and fundamental links between intestinal microbiota and these illnesses, identifying microbial profiles important in the pathogenesis of obesity and CRC may offer a helpful marker for the early detection or prediction of these diseases, and modulating the species composition of these communities may offer an appealing therapeutic alternative. A multifaceted strategy for public health is required to combat the global epidemic of obesity-related cancer risk and progression. Collaborative research projects combining different omics platforms (metabolomics, proteomics, lipidomics, and glycomics) may be able to reveal the main molecular pathways underlying this process, paving the way for the creation of microbiome-based preventative and treatment paradigms. It will, however, be important to distinguish between bacteria and metabolites that are good for health and those that are linked to disease. Furthermore, additional research into the metabolic interactions between cancer cells and the microbiome where the tumor is growing needs to be conducted. Finally, as the role of microorganisms becomes clearer, more research into the mechanisms of action of each microorganism in obesity and associated CRC carcinogenesis may be undertaken. Major microbial variations between healthy and malignant tissue as the disease advances can help identify dysbiosis indicators. Understanding dysbiosis may also help explain why some people with comparable clinical traits experience varying disease progression and therapeutic outcomes. The logical next steps are to identify the causes of these diseases and create better diagnostics and treatments for them. By altering the microbiota through dietary changes, prebiotics, probiotics, symbiotics, postbiotics, FMT, and bacteriophage therapy, it is hoped to move dysbiosis toward eubiosis. Although intriguing, microbiome research has its own set of issues. The study cohorts must be carefully planned, and all pertinent characteristics must be taken into account in statistical analyses, as the composition of the microbiota might vary with geography, age, eating habits, BMI, prescription medications, antibiotics, and pet ownership. Two other crucial factors for microbiome research are the preservation of the original microbiota and the prevention of contamination during sample collection and analysis. Future trials could incorporate a personalized, integrated approach that considers the unique clinical and pathological histories of every patient. This way, the positive effects of gut microbiota-targeted treatments can be maximized while any potential negative side effects may be avoided. 

## Figures and Tables

**Table 1:** Studies demonstrating the role of the gut microbiota and its metabolites in obesity and CRC models.

Disease Model	Outcomes	References
OB-CRC patients	Decrease in butyrate-producing bacteria. Overabundance of opportunistic pathogens. Increased levels of proinflammatory cytokine IL-1β, the deleterious bacterial metabolite TMAO, and gut permeability.	[48]
OB-CRC patients	The α-diversity was higher in patients with colorectal cancer versus controls. The relative abundance of the genera *Enterococcus*, *Capnocytophaga,* and *Polaribacter* was significantly altered with reduced presence of E. faecalis.	[49]
HFD mouse model	HFD promotes CRC by inducing gut microbial dysbiosis, metabolomic dysregulation with elevated lysophosphatidic acid, and gut barrier dysfunction.	[50]
CRC patients	Decrease in butyrate-producing bacteria *Eubacterium rectale*, *Faecalibacterium prausnitzii*.	[51]
CRC patients	Enrichment of potential pathogens. Decrease in butyrate-producing members *Faecalibacterium* and *Roseburia.*	[52]
CRC patients	Increase in *Enterococcus*, *Escherichia/Shigella*, *Klebsiella*, *Streptococcus*, and *Peptostreptococcus*. Decrease in Genus *Roseburia* and other butyrate-producing bacteria of the family *Lachnospiraceae*.	[53]
CRC and colorectal adenomas patients	Altered gut metabolites and microbiota interactions are implicated in colorectal carcinogenesis and can be non-invasive diagnostic biomarkers.	[54]
CRC cellular model	Microbial metabolite deoxycholic acid promoted vasculogenic mimicry formation and EMT through VEGFR2 activation, which further exacerbated intestinal carcinogenesis.	[55]
Mouse model of CRC associated with colitis	Combined dosing of SCFAs (67.5 mM acetate, 40 mM butyrate, 25.9 mM propionate) inhibited tumor formation and reduced colon inflammation.	[56]
CRC mouse and cellular models	*Faecalibaculum rodentium* and its human homologue, *Holdemanella biformis*, produced SCFAs that contributed to control protein acetylation and tumor cell proliferation by inhibiting calcineurin and NFATc3 activation in mouse and human settings.	[57]
CRC cellular model	Greater inhibitory efficacy of butyrate over propionate and acetate against human colon cancer cell proliferation via cell cycle arrest and apoptosis.	[58]
HFD-induced intestinal tumor development in Apc^min/+^ mice.	Butyrate-producing *C. butyricum* exhibited decreased proliferation, increased apoptosis, and suppressed the Wnt/β-catenin signaling pathway. Positively modulated the gut microbiota composition, by decreasing some pathogenic bacteria and bile acid (BA)-biotransforming bacteria and increasing some beneficial bacteria, including SCFA-producing bacteria.	[59]
CRC cellular models	BAs decreased activation of functional farnesoid X receptor (FXR) signaling in CRC cells, promoting colonic carcinogenesis and CRC risk.	[60,61]
CRC cellular models	*Fusobacterium nucleatum* promotes colorectal carcinogenesis by modulating E-cadherin/β-catenin signaling via its FadA adhesin.	[62]
CRC mouse model	*Fusobacteria* generate a proinflammatory microenvironment through recruitment of tumor-infiltrating immune cells, which is conducive for colorectal neoplasia progression.	[63]
Obese humans	Gut microbiota is enriched in *Lactobacillus reuteri* and depleted in *Bifidobacterium animalis* and *Methanobrevibacter smithii*.	[64]
Obese mice model	Obese microbiome has an increased capacity to harvest energy from the diet. Colonization of germ-free mice with an ‘obese microbiota’ results in a significantly greater increase in total body fat than colonization with a ‘lean microbiota’.	[44]
Mouse models	Exercise and butyrate supplement increase butyrate-producing bacteria in the gut microbiota, and increases the production of butyrate, thereby improving lipid metabolism through the butyrate-SESN2/CRTC2 pathway and protecting against obesity.	[65]
Cellular and murine models	*L. paracasei* and *E. coli*, impacted host lipid metabolism in a diet-dependent manner. *L. paracasei* resisted HFD-induced obesity.	[66]
HFD-induced obese mouse model	Decreased fat storage by regulating levels of angiopoietin-like 4 protein (ANGPTL4).	[67]
HFD-induced obese mouse model	Gut microbiome is a critical factor for the anti-obesity effects of Capsaicin (CAP). CAP increased levels of butyrate-producing *Ruminococcaceae* and *Lachnospiraceae*, while it caused lower levels of members of the LPS-producing family S24_7. CAP prevented HFD-induced intestinal barrier dysfunction by inhibiting cannabinoid receptor type 1.	[68]
HFD-induced obese mouse model	SCFA butyrate is effective in alleviating diet-induced obesity through activation of the ARβ3-mediated lipolysis in the epididymal white adipose tissue. SCFA acetate reduces appetite via a central homeostatic mechanism.	[69,70]

## Data Availability

Not applicable.
